# Clinic Atlanta Medical College

**Published:** 1875-11

**Authors:** A. W. Calhoun

**Affiliations:** Professor of Diseases of the Eye and Ear


### Clinic Atlanta Medical College.

A. W. CALHOUN, M.D., Professor of Diseases of the Eye and Ear.

(*Continued from page* 398 *of October Journal.*)

Case XXII.—*Foreign Body in the Cornea—Removal.*—J. T., white, æt. twenty-five years, Atlanta, Ga., presents himself with a foreign body (a piece of iron) deeply sunken into the corneal tissue, just in front of the pupil. It gives intense pain, which, with the inflamed condition of the eye, renders its immediate removal necessary.

Where the foreign body lies superficial, not imbeded, a magnet will often succeed in bringing it away, by attracting and holding it sufficiently firmly to withdraw it from its seat. Prof. Hasner, of Prague, has invented a little instrument, which he calls "the magnetic forceps," having the combined advantages of an ordinary pair of forceps and a magnet, the two rough points of the instrument being so constructed as to have the magnetic power. Unless the foreign body lies very deep, this instrument answers a very admirable purpose, for at the same time you grasp the body, the influence of the magnet is exerted, and you succeed in extracting it.

As a rule, foreign bodies imbed themselves rather deeply, and when so the quickest and most humane mode of treatment is to open the lids by means of an eye speculum, and having fixed the ball with forceps, to loosen and root out, so to speak, the body with an ordinary cataract needle, or with a grooved needle. Those of you who know from experience the difficulties to be met, with in removing such bodies from the eye, on account of the extreme sensitiveness of the organ, will appreciate that plan, which enables you to succeed as rapidly and with as little pain to the patient as possible.

In this case the lids are held apart and ball fixed and the body scratched out as mentioned above. The eye was bandaged to keep the lid quiet over the abraded cornea, and on the following day the patient dismissed as cured.

CASE XXIII.—*Myringitis or Inflammation of the Membrana Tympani* (*Ear Ache.*)—C. M., colored boy, æt. nine years, Atlanta. The patient has been exposed to bad weather and has taken cold, and now complains of intense pain (worse at night) in right ear, producing high fever, restlessness, etc. Further than a sensitive and slightly swollen condition of the parts around the ear, no outward signs of inflammation are present, but upon examination with the mirror and ear speculum, with which the light is thrown into the ear, the tympanum is found to be immensely congested, filled with little capillary blood vessels, having lost all of its natural ashy gray appearance, and the adjacent parts of the external canal are also inflamed and swollen. Of course there is but little ability to hear from this ear. To this condition of things the common term of "ear ache" is applied, but not to this alone, for inflammations of another character, such as abscesses in the external ear, etc., bear also the same title.

In such cases active treatment is necessary; for instance we will here abstract blood locally from the temple, or mastoid process, or both, by means of leaches or cupping, to the extent of five or six ounces. If this does not relieve the pain, fly blisters will be applied to the same parts. We will also give a calomel purge. As a local application, one grain of morphine in an ounce of water, to be poured tolerably warm into the ear every half hour or hour, will often afford quick relief. If you apply poultices, leave the entrance into the ear free, so as not to allow the heat to be collected and held in contact with the drum. This would keep it too much congested. Glycerine and morphine will also be found to answer well, but bear in mind to have the fluids milk warm upon pouring them into the ear. Such treatment will usually reduce the inflammation, which, when left alone, or badly treated, not unfrequently ends in perforation of the drum, suppuration of the middle ear, and even some times passes through the thin layer of bone separating the middle ear from brain, and produces inflammation of the membranes surrounding the brain substance, (meningitis.) If necessary, an opiate must be given the child to insure rest and sleep. For the present this mode of treatment will be carried out.

CASE XXIV.—*Suppuration of the Middle Ear (Otorrhœa.) Treatment with Alcohol.*—M. W., colored cook, æt. fifty years, Atlanta. The patient has a constant fœtid discharge from the right ear, which has existed for several years. Upon examination we find the discharge comes from the middle ear and empties itself through an opening in the membrana tympani. These chronic otorrhœas are very stubborn, but succumb usually to the proper treatment constantly kept up. I bring this case before you to demonstrate the efficacy of the alcohol treatment, which acts in many cases most surprisingly. Before applying it, the ear must be thoroughly cleansed by syringing with milk warm salt water, and afterwards dried as nearly as possible. This latter is important, else oftentimes a sufficiency of water is left behind to dilute the medicine too much. If you have reason to suppose this will not be properly carried out, it will be better to use a moistened camel's hair pencil, and swab out the ear, and thus cleanse it of matter. After this is done, fill the ear with the pure undiluted alcohol, and let it remain in for five or ten minutes. The first effect of it is a sharp burning feeling, *which is only momentary*, and then a very pleasantly warm sensation follows. At the end of five or ten minutes, turn the head to one side to empty the fluid, and dry the ear, and put in a piece of cotton. Make this application twice daily, but keep the ear cleansed oftener, if necessary, according to the copiousness of the discharge.

I have known otorrhœas, existing for many years, cured in comparatively a short time by this treatment, and can recommend it strongly to you for trial. There are cases, of course, where other remedies must be resorted to, or where they should be used alternately with the alcohol. In cases of polypus, or diseased bone, of course, this is not applicable.

We will give it a trial in this case, and watch the result.

CASE XXV.—*Deafness from an Acute Inflammation (with closure) of the Eustachian Tube and Middle Ear. Recovery of Hearing.*—S. G. B., white, æt. twenty-five, Atlanta. The patient has taken a severe cold, and the inflammation has extended from the throat along the eustachian tubes into the middle ear, causing the lining membrane of the tube to swell to such an extent as to prevent the passage of air into the middle ear. Through the semi-trasparent drum, the swollen and inflamed condition of the mucous membrane of the middle ear can also be seen. Within the last few days the hearing has gradually diminished, until only loud conversation is audible. There is a constantly dull, aching sensation, and a fullness in the ears, and a continual roaring in the head, day and night, without cessation. Occasionally, as the patient opens the mouth widely, or blows his nose, "the ears open, and for a moment the hearing is quite good," but soon the roaring and deafness again make their appearance. Upon introducing the catheter into the eustachian tubes through the nose, and throwing air into the ear, the patient at once hears better, the roaring ceases, and most of the unpleasant symptoms disappear, but reappear after several hours. This treatment was kept up once daily for two or three weeks, injecting at the same time into the middle ear, through the catheter, a weak solution of iodide potassium, to cause a diminition of the inflammation and swelling of the mucous membrane lining the parts. Occasionally this was alternated with a solution of hydrate chloral, or the fumes of chloroform, for the same purpose. At the end of three or four weeks every unpleasant symptom had vanished, and the hearing was fully restored.

